# The Effect of Cognitive Load on Information Retention in Working Memory: Are Item Order and Serial Position Different Processes?

**DOI:** 10.3390/brainsci15111179

**Published:** 2025-10-30

**Authors:** Davide Baggini, Paola Ricciardelli

**Affiliations:** Department of Psychology, University of Milano-Bicocca, 20126 Milan, Italy; d.baggini@campus.unimib.it

**Keywords:** working memory, cognitive load, time-based resource sharing, serial order, information storage

## Abstract

Background/Objectives: A central question in cognitive neuroscience is how information is transferred from working memory to long-term memory, and what factors influence this process. This study aimed to explore the role of cognitive load in the consolidation of information into long-term memory within the framework of the Time-Based Resource Sharing model of working memory. Methods: An exploratory study was conducted using a reading digit span task with delayed response, in which cognitive load was manipulated through Hebb repetition learning. Results: An improvement in the ability to remember the order of the elements was found with the decrease in cognitive load, consistent with the hypothesis that the transfer of information to long-term memory occurs during the maintenance process and involves cognitive load. However, no improvement in the recall of the total number of elements emerged, suggesting that different mechanisms and factors are at play in the process of information transfer. Conclusions: These findings shed new light on the complexity of interactions between working memory and long-term memory, paving the way for further systematic investigations into the nature of mechanisms responsible for transferring information from the former toward the latter.

## 1. Introduction

The human cognitive system constantly collects, processes, and stores information. Most of this information is usually only maintained for a few seconds, but it can also persist for days, months, or even over the whole lifetime in long-term memory. In this study, we focused mainly on a specific model of working memory, the Time-Based Resource Sharing (TBRS) model [1]. One limitation of this model is that it does not properly integrate the relationship between working memory and long-term memory and how information is transferred from the former to the latter and vice versa. Since it is difficult to conceive that working memory has no functional connections with long-term memory, especially in cases where information depends on past experience, we decided to explore such a relationship in this study. The present research aimed to shed some light on the process underlying how information stored in working memory for a brief time is transferred to long-term memory.

The working memory theoretical construct has progressively replaced the outdated short-term memory concept to account for the retention and active processing of information in the short term [2]. It has been realized that the conceptualization of short-term memory was a conception of information storage that was too passive and did not take into consideration many cognitive processes, leading to poor predictions of real-world phenomena. Working memory, instead, is a richer cognitive construct that describes how immediate memory actively processes information and maintains it in the short term to deal with complex everyday tasks, such as, for example, problem solving and language comprehension, that require many different processes, constraints, and interactions between these processes [3]. One of the most widely accepted models of working memory is the multi-component model proposed by Baddeley and Hitch, in which working memory is described as a collection of sub-structures with different roles rather than a unitary system [4,5,6].

The initial version of the multi-component model faced a major limitation: it did not account for the relationship between working memory and long-term memory. Evidence from memory span tasks highlighted this gap. While unrelated words yield a span of about five to six items, grammatically structured sentences extend the span to approximately fifteen [7]. Such findings challenged the model, as this exceeds the phonological loop’s capacity. The increased span arises from chunking based on syntactic and semantic relations, processes that crucially depend on knowledge stored in long-term memory. This highlighted the need to explain how working memory draws upon and integrates long-term representations and raised the question of how working memory can access and use information stored in long-term memory. Supporting evidence comes from the visuo-spatial domain: processing familiar images is not limited to the visuo-spatial sketchpad but is facilitated by the amount and quality of long-term memory knowledge [8].

To address this limitation, a crucial component was introduced to the model: the episodic buffer [5]. This system can hold about four chunks of multidimensional information, integrating inputs of different types (visual, semantic, and verbal) originating from long-term memory, working memory, or sensory sources. Since these inputs must be bound into coherent representations, Baars [9,10] proposed that consciousness serves as a mental workspace, where streams of information converge and are integrated into unified objects and events.

In its initial formulation, the episodic buffer was conceived as an active system under central executive control, capable of integrating separate elements into coherent representations, for instance, combining words into sentences or uniting color and shape into object concepts [5]. Allen, Baddeley, and Hitch [11] tested this by overloading the buffer with concurrent tasks, finding that while executive demands impaired general performance, they did not strongly disrupt integration. This led Baddeley [6,12] to argue for specialized subsystems managing different types of integration, such as visual or linguistic. Incorporating the episodic buffer thus helped bridge the gap between storage-focused accounts, such as Baddeley and Hitch’s model, and attention-centered perspectives, such as Cowan’s [13,14]. This shift stimulated research on how working memory interacts with long-term memory, particularly in relation to visual attention and memory [15,16].

The multi-component model is not the only model of working memory, and alternative models, each based on different assumptions and trying to describe a different aspect of working memory, have been proposed in recent decades (e.g., ref. [17,18]).

TBRS is a working memory model developed to explain the mechanisms underlying the simultaneous processing and maintenance of information in working memory [1]. In relation to Marr’s levels of explanation [19], one of the main differences between Baddeley’s multi-component model and the TBRS model is that the former tries to describe working memory at a computational level, while the latter does it from a representational level, allowing quantitative predictions by accounting for the decay of information in the short term as a function of time. In the TBRS model, processing and maintaining information in working memory are two distinct activities performed within the same system. Processing is a function that results in the production of a new piece of information following a computation, whereas maintenance refers to the ability to hold and retrieve information after it has been encoded. The authors designed the TBRS model based on four main assumptions: (i) Processing and maintaining information are active processes that both require attention, which is a limited resource. The two activities share the same pool of attentional resources. (ii) A central bottleneck only allows the focus of attention to operate on one process at a time, so sharing attention is time-based. Attentional focus, therefore, must alternate between processing and maintaining information, since it cannot be split. (iii) When attention is switched away from a to-be-recalled piece of information, its memory trace suffers from decay due to the passage of time (i.e., time-related decay). To avoid such a decay of information, refreshing the memory trace is necessary, but this action requires attentional resources. (iv) It is assumed that there are periods of time during which attention is totally engaged in information processing, hence preventing memory traces from being refreshed. Thus, attention sharing is achieved through rapid and frequent switching between processing and maintaining multiple memory traces during task completion. Because of these several alternating activities that depend on attentional resources, the functioning of working memory is time-based.

**Figure 1:**
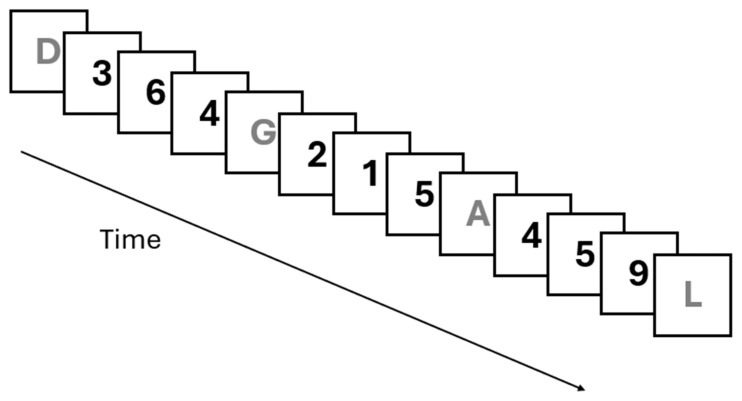
Example of the reading digit span task. Between the presentation of each target (the letters), a series of digits to be read aloud is displayed in sequence as an interference task. At the end of the trial, participants are asked to recall the targets in the correct order.

According to the TBRS model, memory performance mostly depends on the cognitive load of the task. In fact, according to the TBRS model, no task is intrinsically easy or hard. What makes a task easy or hard to perform is the cognitive load level of the task, given by the specific circumstances in which it is carried out. In this model, cognitive load represents the proportion of time during which the focus of attention is captured by processing operations and, therefore, it is not available for maintaining memory traces in an active state. It reflects the time taken by processing activities over the total time available for the task. In other words, the cognitive load of an activity increases proportionally with the ratio between the time used for processing and the total time available for the task, and it is given by the following formula:

CL = aN/t

where *N* is the number of processing steps, *a* is their average duration, and *t* is the total amount of time available for performing the task. To test the TBRS model, the authors used the reading digit span task [1]. This experimental paradigm consists of the serial presentation at a constant pace of to-be-recalled targets (i.e., letters), each followed by a series of stimuli to be processed (i.e., a series of digits) that compete for the same resources. Participants must process the interfering stimuli during the presentation (in this example, sum the numbers as soon as they appear) and then recall the targets at the end of the presentation (see Figure 1).

**Figure 2:**
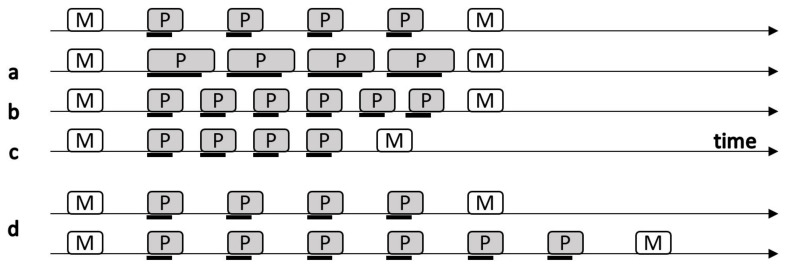
Variations in cognitive load. The first row depicts successive screens on a computer-paced reading digit span task in which each memory item M is followed by a series of items to be processed P. Black lines beneath P items represent the portion of time during which processing occupies attention. Row **a** illustrates the increase in cognitive load resulting from an increase in the duration of attentional request (parameter *a* in the cognitive load formula) while the number of processing steps *N* (number of items to be processed) and the total time *t* allowed to perform them remain constant. Row **b** illustrates the increase in cognitive load resulting from an increase in the number of processing steps *N* while parameters *a* and *t* remain unchanged. Row **c** illustrates the increase in cognitive load resulting from a reduction in t while *N* and *a* remain constant. By contrast, increasing the number of processing steps performed at a constant pace leaves cognitive load unchanged (row **d**). (Image and caption adapted from Barrouillet & Camos, 2014 [21]).

By manipulating the proportion between the free time (i.e., time not used to process new information) and the time occupied to process the interfering items, it is possible to vary the cognitive load of the task itself (see Figure 2 for a graphical example). During the last few years, a remarkable amount of literature has been produced to support predictions of the TBRS model [20]. As predicted by the TBRS model, memory performance is inversely proportional to the cognitive load of the task. This suggests that memory traces can only be refreshed during free time (i.e., when attention is not captured by the processing of interfering items).

Some models conceptualize working memory as an activated subset of long-term memory under attentional focus [13,14,18,22,23]. In contrast, the time-based resource sharing model treats them as distinct systems, emphasizing differences in representational maintenance. Working memory traces are transient and subject to time-related decay unless actively refreshed, whereas long-term memory representations are more stable, decaying primarily through interference from new content generated in working memory.

A phenomenon that is usually considered a reflection of the gradual transfer of information from working memory to long-term memory is the so-called Hebb repetition learning effect [24,25,26,27,28]. Hebb repetition learning is a well-known sequential learning paradigm that is assumed to rely on the same cognitive resources as word-form learning, the ability to learn the phonological forms of newly presented words [29,30]. A classic Hebb repetition learning paradigm consists of the immediate serial recall of presented sequences of items (usually characters or phonemes). The sequences can be either completely new (called filler sequences) or already presented in previous trials (called Hebb sequences). In this task, when recalling Hebb sequences, performance usually improves compared to when the filler sequences have to be recalled [31]. This is the case regardless of whether participants are aware of having seen the sequence before, as these repeated sequences become consolidated in long-term memory. This effect raises questions about how the TBRS model accounts for such a long-term learning effect. The primary aim of the present study was to investigate the mechanisms underlying information transfer from working memory to long-term memory and to assess whether the TBRS model can also account for long-term retention of information. In particular, we focused on the role of cognitive load in the information storage process.

There is evidence in the literature suggesting how working memory performance is bound to a limited amount of available resources (for example, ref. [32,33,34,35,36]) but there is also evidence that, through repetition and practice, it is possible to reduce the involvement of working memory processes, since the process became automatic, thus requiring less or very little attentional resources [37]. It is also possible to improve performance in working memory tasks after training [38], suggesting that fewer resources might be required to perform the same task when gaining experience.

Baddeley and colleagues conceptualize the relationship between working memory and long-term memory as complex, flexible, and interactive [39]. The authors view working memory as a bridge between cognition and action, capable of drawing on multiple inputs and processing levels, while acknowledging that not all information must pass through it. Importantly, they argue that working memory necessarily involves the activation of long-term memory, framing this claim as a challenge for future research rather than a definitive explanation. In the time-based resource sharing model, consolidation into long-term memory is assumed to depend on the repeated maintenance of traces in working memory, with more frequent reconstruction leaving stronger imprints. However, empirical evidence for this account remains limited.

The present study aimed at answering the following research question: Does the cognitive load of an activity have an impact on the amount of information transferred from working memory to long-term memory? Our experimental hypothesis was that cognitive load influences the amount of information that can be transferred from working memory to long-term memory. More precisely, the higher the cognitive load, the less information it is possible to transfer. We expected such an effect because a high cognitive load task would require a greater amount of resources to be allocated to perform the task, thus leaving fewer resources to transfer information to long-term memory than a low cognitive load task.

## 2. Materials and Methods

### 2.1. Participants

To determine the sample size, we relied on a classical study by Postman and Phillips [40] that used a similar experimental paradigm. Since the effect sizes were not reported in the original article, we opted to double their original sample size. This approach aimed to ensure more stability and precision in parameter estimates and to reduce the risk of underpowered statistical tests, while maintaining continuity with prior literature. This rationale is a reasonable pragmatic strategy in replication and paradigm-extension studies when prior effect size information is unavailable.

A total of 70 participants, recruited among students at the University of Milano-Bicocca, voluntarily took part in the study. A group of 35 were randomly assigned to the experimental condition (Women = 20, men = 15, age M = 28, SD = 8.5) and the remaining 35 to the control condition (Women = 22, men = 13, age M = 24.4, SD = 4.2). All participants had normal or corrected-to-normal vision, and none of them had neurological or learning diseases. All participants consented to data treatment and received university credits for their participation.

The study was considered minimal risk (as defined by the National Research Council of the Academies of Science and, as such, was approved by the Committee for Research Evaluation of the Psychology Department of the University of Milano-Bicocca (protocol number N. RM-2023-619 approved on 2 August 2023) and was conducted in accordance with the Helsinki treaty.

### 2.2. Materials and Stimuli

The authors of the TBRS model have widely used the reading digit span task for assessing their model; for this reason, we decided to stick to this paradigm even though, in the present study, it has been modified by pairing it with an n-back task.

The reading digit span task consists of a series of stimuli presented at a fixed pace on a computer screen. Some of the stimuli are targets that must be maintained in memory for recall, while the others are just distracting stimuli that must be processed but not remembered. In our study, the targets and the distractors for the reading digit span task were both letters that participants were asked to read aloud as soon as they appeared on the computer screen. The difference between the two categories of stimuli was that targets were depicted in red, while distractors were depicted in blue. Trials always began with the presentation of a fixation cross, followed by the first target. Four targets were presented per trial, and four distractors followed each target. Stimuli were presented at a constant pace of one per second. Participants were instructed to read aloud all the characters but only remember the red ones. In the response phase, the word “REPLY” was displayed on the computer screen, and participants were asked to type on a keyboard the red letters they remembered in the correct order they were presented.

In order to delay the response of the reading digit span task, an n-back task was added to each trial between the stimuli presentation and response phases of the reading digit span task.

The stimuli used in both the reading digit span task and the n-back task were Western upper-case letters depicted in different colors (red or blue for the reading digit span task and black for the n-back task). The stimuli were presented on a 17′ monitor, and occupied approximately 2.5° of visual angle.

### 2.3. Procedure

The task was administered in a quiet room with controlled artificial light. To avoid any potential distraction, participants were asked to turn off all their electronic devices. The whole task was programmed and presented in Matlab [41] using the Psychophysics Toolbox extensions [42,43,44].

**Figure 3:**
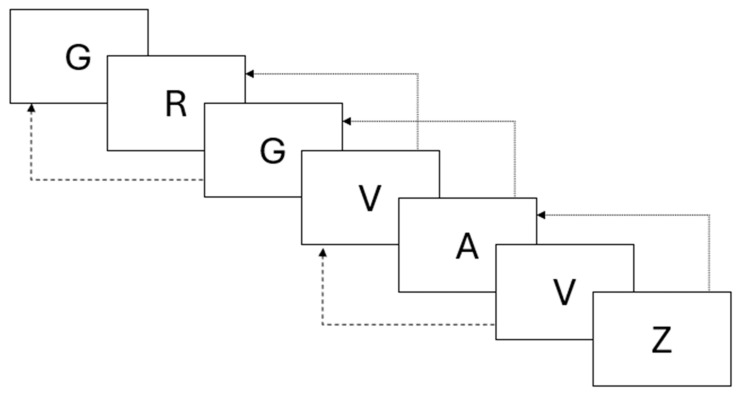
Example of the n-back task. Stimuli are presented one at a time, and participants must decide whether the currently displayed one was the same or not as the *n*th before. This example presents a 2-back task, meaning that participants must compare the currently displayed character with the character presented 2 positions before; dashed arrows represent “match” responses, and dotted arrows represent “mismatch” responses. Note that in n-back tasks, the first n stimuli (in this case, 2) do not have to be compared to anything, but they must be maintained in memory to be compared with the following stimuli.

The experiment was structured as a between-subjects study. Participants were asked to perform a reading digit span task with a delayed response, meaning that between the stimuli presentation phase and the response phase, they were asked to perform a 2-back task. The n-back task is a commonly used paradigm in working memory studies. It is a demanding task in which information in working memory must be constantly maintained and updated. It consists of a series of stimuli presented one at a time, and participants must decide whether the currently displayed stimulus matches the *n*th previous stimulus. For example, in a 2-back task, the participant must decide whether the current stimulus is the same as or different from the stimulus presented 2 steps before (see Figure 3 for an example).

**Figure 4:**
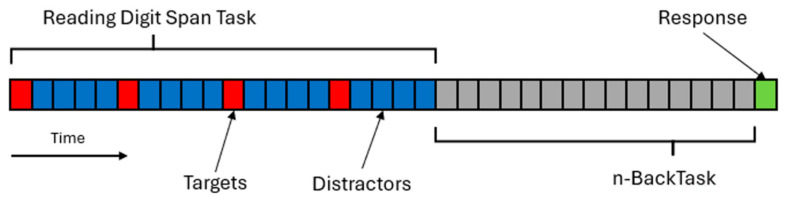
Schematic representation of the progression of a single trial of our experiment. Each cell represents a single stimulus presented on the screen. Red cells are reading digit span task targets, blue cells are reading digit span task distractors, gray cells are n-back task stimuli, and the final green cell represents the response phase.

**Figure 5:**
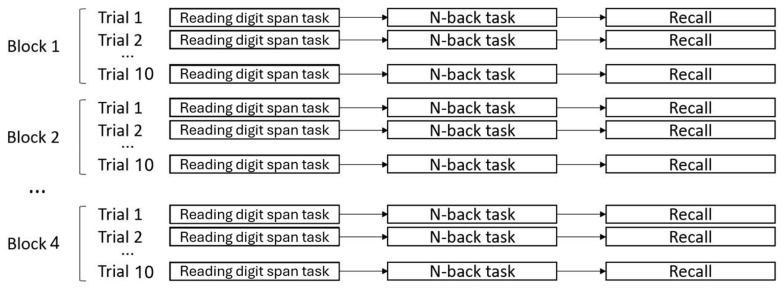
Representation of the experimental paradigm structure. Each trial was composed of three phases: a reading digit span task phase, an n-back task phase, and a recall/response phase. Within each block, the series of distractors used in the reading digit span task remained identical across trials until the end of the block.

The stimuli used in this study for the 2-back task were characters depicted in black, and participants were asked to decide whether the currently displayed character was the same or not compared to two characters before and give a response by pressing one of two keys on a QWERTY keyboard (Z for “same” and M for “different”). A 2-back series of fifteen stimuli was presented after each reading digit span task presentation. The 2-back task was self-paced, meaning that every stimulus appeared on the screen as soon as the answer for the previous stimulus was given. However, participants were instructed to answer as fast and accurately as possible without taking any breaks between stimuli or between the two tasks. Figure 4 represents the structure of a single trial. The general structure of the task is presented in Figure 5.

Before the experiment began, participants signed the informed consent form and were then presented with a training phase to familiarize themselves with the task. They first made three practice trials with the reading digit span task alone, then three practice trials with the 2-back task alone, and finally three practice trials with both tasks in succession as described above. The experiment was divided into four blocks of 10 trials each, for a total of 40 trials. In the experimental condition, distractors (i.e., blue letters) remained the same across all trials within a block, while targets (red letters) and 2-back task stimuli were always randomly generated; therefore, targets were different in every trial. In the control condition, on the other hand, all the stimuli (i.e., targets and distractors) were randomly generated for each trial, meaning that they were always different. Participants were not aware of the division of the task into blocks in the experimental condition.

Accuracy was measured for both the number of reading digit span task targets correctly recalled and their sequential order reported in the response. For measuring the presence of targets in the participant’s responses, the response string was compared to the target string presented in the trial, and for each character in the response that was also in the target array, a score of 1 was assigned to the trial; 0 otherwise. To check for the correctness of their position, the two arrays of targets and response were compared, and only the characters in the response that were reported in the same position as in the target array were assigned a score of 1, and 0 otherwise. In this way, each trial has a score between 0 and 4 for both the presence of targets in the response and for their absolute position. Four targets were presented in each trial.

## 3. Analysis and Results

**Figure 6:**
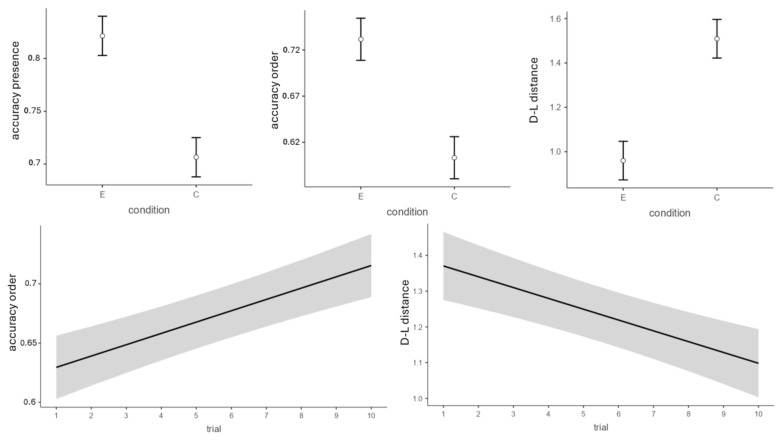
The **top row** represents the average accuracy for the two conditions for the three dependent variables considered. The **bottom row** represents the effect of trial on accuracy for remembering targets in the correct position and on the Damerau-Levenshtein distance. Only the first and last trials are considered. Bars and gray areas represent standard error.

First, we compared the first and last trials of each block, since they represent the highest and lowest cognitive load trials, respectively. A series of analyses was conducted using a linear mixed model to test performance on different parameters. In the first analysis, only the presence of a character in the response was tested, regardless of the correctness of its position. The dependent variable was the participants’ accuracy in remembering the target items. Condition, trial number, and block number were set as fixed effects, while subjects were clustered as random effects in order to account for interpersonal differences in performance. Estimates have been obtained through the REML method. Results showed a significant main effect of condition (*β* = 0.49, *SE* = 0.2, *t*(58.3) = 2.41, *p* = 0.019) and block (*β* = 0.09, *SE* = 0.04, *t*(389) = 2.3, *p* = 0.022) on accuracy, with an overall higher performance in the experimental condition compared to the control condition, and an increase in performance across blocks (Figure 6).

The same analysis was conducted using the accuracy in remembering the correct number of target characters in the correct position as a dependent variable. A significant main effect of condition (*β* = 0.54, *SE* = 0.24, *t*(62.4) = 2.28, *p* = 0.03), trial number (*β* = 0.03, *SE* = 0.01, *t*(389) = 2.93, *p* = 0.004), and block (*β* = 0.17, *SE* = 0.05, *t*(389) = 3.42, *p* < 0.001) emerged. The direction of the effects was coherent with those reported in the previous analysis, with a greater overall accuracy in the experimental compared to the control condition, an increase in accuracy across blocks, and an increase in accuracy across trials (Figure 6).

As an additional measure of accuracy, we computed the Damerau-Levenshtein distance between the correct responses and the responses given by participants, and we performed the same analysis using the Damerau-Levenshtein distance as the dependent variable. The results of this last analysis were in line with the previous ones reported. In fact, a significant main effect of condition (*β* = −0.56, *SE* = 0.23, *t*(64) = −2.42, *p* = 0.018), trial number (*β* = −0.03, *SE* = 0.01, *t*(389) = −2.38, *p* = 0.017), and block (*β* = −0.14, *SE* = 0.05, *t*(389) = −2.78, *p* = 0.006) emerged once again (Figure 6).

To complement the earlier comparison between the first and last trials, the following analyses were conducted on the complete set of 10 trials per block. The same analyses have been conducted, with the only difference being that all the trials were included.

**Figure 7:**
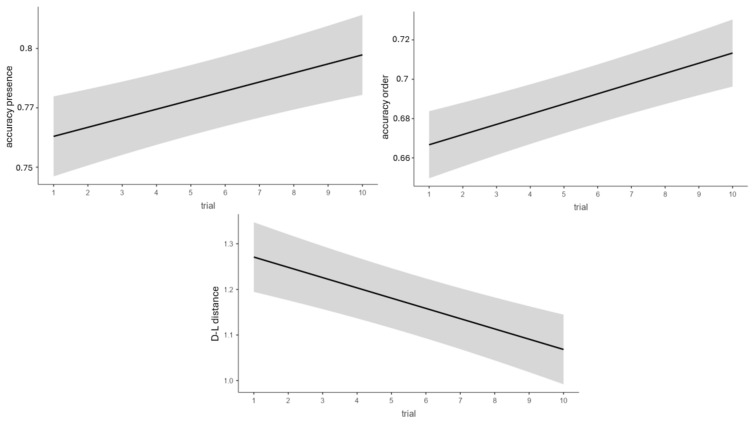
Effect of trial on accuracy for remembering the presence of targets regardless of their order, on accuracy in remembering targets in the correct position, and on the Damerau-Levenshtein distance. All the trials are considered. Gray areas represent standard error.

**Figure 8:**
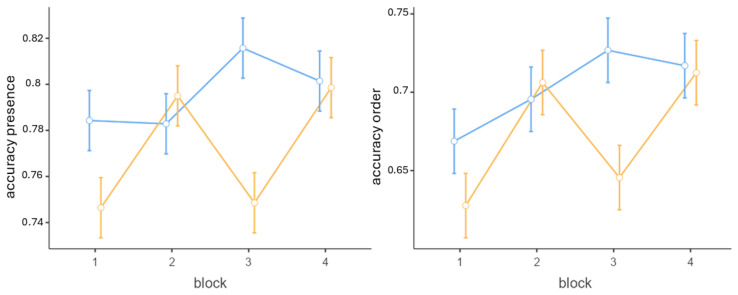
In response to a reviewer’s request, we also report the average accuracy for each block divided by condition. The blue line represents the experimental condition; the yellow line the control condition. All trials have been considered. The plot on the left represents the accuracy in remembering the presence of targets, and the plot on the right represents the accuracy in remembering their order. Overall, in the experimental condition accuracy is higher than in the control condition. The experimental condition shows an improvement in performance across trials compared to the control condition. Bars represent standard error.

When considering the accuracy in remembering the presence of the target characters as a dependent variable, a significant main effect of trial (*β* = 0.01, *SE* < 0.00, *t*(2181) = 2.11, *p* = 0.035) and block (*β* = 0.04, *SE* = 0.01, *t*(2181) = 2.61, *p* = 0.009) emerged (Figure 7 and Figure 8).

When the dependent variable was the accuracy in remembering the targets in the correct position, a significant main effect of trial (*β* = 0.02, *SE* < 0.00, *t*(2181) = 2.82, *p* = 0.005) and block (*β* = 0.07, *SE* = 0.02, *t*(2181) = 3.13, *p* = 0.002) emerged (Figure 7 and Figure 8).

When the dependent variable was the Damerau-Levenshtein distance, once again, a significant main effect of trial (*β* = −0.02, *SE* < 0.00, *t*(2181) = −2.58, *p* = 0.01) and block (*β* = −0.04, *SE* = 0.02, *t*(2181) = −2.1, *p* = 0.035) emerged (Figure 7 and Figure 8).

The average number of targets correctly recalled by participants was 2.96 (*SD* = 0.69), with skewness = −0.98 (*SE* = 0.39) and kurtosis = 0.23 (*SE* = 0.77) in the experimental condition, and 2.88 (*SD* = 0.65), with skewness = −0.61 (*SE* = 0.39) and kurtosis = −0.38 (*SE* = 0.77) in the control condition. The average Damerau-Levenshtein distance was 1.35 (*SD* = 0.81), with skewness = 0.77 (*SE* = 0.39) and kurtosis = −0.3 (*SE* = 0.77) in the experimental condition, and 1.5 (*SD* = 0.77), with skewness = 0.55 (*SE* = 0.39) and kurtosis = −0.46 (*SE* = 0.77) in the control condition.

**Figure 9:**
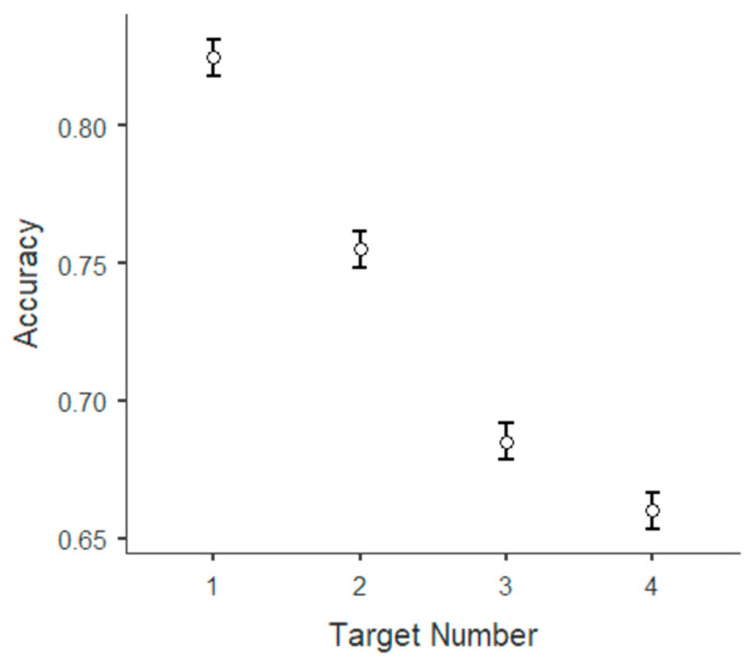
Accuracy in remembering the presence of a target. Accuracy is expressed as the proportion of responses that correctly included the target. On the *x*-axis is represented the position of the target in the presentation. Bars represent the standard error.

Finally, we analyzed recall accuracy as a function of target position during presentation within each trial (i.e., whether the target appeared first, second, third, or fourth). A 2 (conditions) by 4 (targets) ANOVA revealed significant main effects of target position (F(3, 11,192) = 86.05, *p* < 0.001, η^2^p = 0.023) and a significant condition x position interaction (F(3, 11,192) = 4.88, *p* = 0.002, η^2^p = 0.001). A post hoc comparison between conditions for each target revealed that the effect of condition alone was non-significant (Figure 9).

## 4. Discussion

In the present study, we tested the hypothesis that cognitive load affects the amount of information that can be transferred from working memory to long-term memory. We expected that a higher level of cognitive load would imply a lower amount of information stored in long-term memory due to a smaller amount of cognitive resources available for information transfer. The results partially confirm our hypothesis, since we found a significant difference in performance between the experimental and control conditions when considering only the first and last trials (i.e., the ones with the highest and lowest cognitive load, respectively), with the average performance in the experimental condition (where cognitive load decreases across trials) being higher. What was unexpected was that the difference in performance given by the trial number was only observed for remembering the order of targets, but not for their presence in the response. In other words, participants improved their performance in remembering the order of presentation of the reading digit span task’s targets, but not in remembering a higher number of targets.

The reason for maintaining the same series of distractors between trials in the experimental condition in our experiment was to decrease the cognitive load of the task at each trial. Since the series was already encountered in previous trials within the same block, it became easier to process the information at every new trial due to Hebb repetition learning. By dividing the whole experiment into four blocks, we observed the effect of gradually decreasing the cognitive load on task performance four times per participant. In fact, thanks to Hebb repetition learning, cognitive load gradually decreased at each trial, but it returned to the starting level at the beginning of a new block of trials since the series of distractors was utterly new. In contrast, in the control condition, distractors were always new at each trial, resulting in a constant level of cognitive load across trials.

In the present study, the rationale behind the decision to use a delayed response paradigm was derived from the assumptions of the TBRS model itself. Specifically, the TBRS model has been widely tested using the reading digit span task and other similar tasks to assess working memory. However, in our study, we were interested in investigating the process of information transfer toward long-term memory. The 2-back task had the purpose of occupying as many cognitive resources as possible and overwriting the content of working memory. According to the TBRS model, information maintained in working memory is refreshed only during periods when no other information is being maintained or processed. Otherwise, it decays as a function of time. Adding an n-back task between the presentation of the reading digit span task and the response phase forced participants to constantly maintain and update new information that was irrelevant to the reading digit span task. In this way, it would be theoretically impossible for participants, according to the TBRS model, to actively maintain the reading digit span task targets in working memory until the response phase. Therefore, the high demand for attentional resources and the delay created by the n-back task should have interfered with the maintenance process of targets enough to observe the effect of our interest.

An additional element that made it more difficult for participants to actively maintain targets in working memory was that they were instructed to read all the characters aloud as soon as they appeared on the screen. This ensured that they were paying attention to and processing all the stimuli and prevented them from refreshing the whole target series through articulatory rehearsal or subvocal repetition. This simple request to read aloud all the characters reduced the time available to participants for processing the targets and maintaining them available for recalling, since it increases the overall cognitive load of the task.

As mentioned above, in the experimental condition, the series of distractors in the reading digit span task always remained identical between trials within each block. In contrast, in the control condition, they were always randomly generated for each trial. This repetition of stimuli in the experimental condition allowed us to gradually decrease the cognitive load of the task by taking advantage of the Hebb repetition learning effect described previously. Thanks to this learning effect, it became easier for participants to process the distractors at every new trial within a block since they had already processed the same series in the previous trials. In other words, they needed a lesser amount of cognitive resources to process the distractors, and therefore, they had a greater amount of resources available to process the targets in the same time frame. According to the TBRS model, this would reflect in a change in the time proportions for processing/maintaining information in the reading digit span task: as trials progress, distractors are processed faster, and more time is available to process and maintain the targets [1]. At the end of the reading digit span task presentation, targets should have been better maintained in late trials compared to early ones. However, the long delay and the significant consumption of cognitive resources caused by the n-back task presented after each trial of the reading digit span task should have led the target memory traces to completely or almost completely decay [45]. This means that, according to our experimental hypothesis that the higher the cognitive load, the less information it is possible to transfer from working memory, if target information has been successfully maintained, it should not have been stored in working memory, but likely during the maintaining phase in working memory, it has been transferred to another structure functionally similar to long-term memory or, possibly, long-term memory itself, capable of holding information but where active maintenance of information is not required or, at least, is less demanding. This process, as expected, should manifest in a gradual improvement in performance across trials within each block. On the other hand, since in the control condition distractors were always different, the cognitive load did not change across trials, and no improvement was expected, except for the generic effect of practice. In other words, the expected finding was an improvement in accuracy when comparing the first (i.e., the highest cognitive load) and the last (i.e., the lowest cognitive load) trial of each block, as a consequence of Hebb repetition learning. In fact, in the first trial of each block, all the stimuli were new, but in trial 10, the series of distractors had already been encountered nine times. Specifically, according to our experimental hypothesis, we expected to find an increase in performance between trials 1 and 10 in the experimental condition group but not in the control group. Indeed, our results show a difference between the groups for the number of items remembered, but no difference was observed between these two trials. Despite not being exactly what we expected, this result is still partially in line with our hypothesis, since participants in the experimental group performed better than the control group, possibly due to the lower overall cognitive load of the experimental condition.

When examining accuracy for target order, we found a significant main effect of condition, trial, and block, although the main effect of condition was only observed when the first and last trials alone were considered for each block. Performance improved from the first (high cognitive load) to the last (low cognitive load) trial within each block, in line with our prediction, suggesting that a lower level of cognitive load allowed for better processing of target information. These results indicate that participants gradually became better at remembering the order of presentation of targets with a decrease in cognitive load. However, they were not able to remember a greater number of items.

As an additional measure of performance, we computed the Damerau-Levenshtein distance between the correct and the given responses. This value represents the edit distance between two strings, expressed by the number of steps required to obtain the correct response starting from the given one, where each step can be the insertion, deletion, substitution, or transposition of characters. The analyses conducted on this metric confirmed the results reported above.

To summarize, a positive effect of the progressive number of trials on accuracy for remembering the order of targets was observed. In line with the main hypothesis, accuracy gradually increased with the number of trials performed within each block. The decrease in cognitive load through trials led to an improvement in performance in remembering the order, but not the total number of targets. Interestingly, if it is true that a greater amount of available cognitive resources is used to encode information in long-term memory, our results would suggest that encoding a series of elements and encoding their order can be two distinct operations.

A possible alternative explanation for not having observed any improvement in remembering the total number of targets can be the presence of a ceiling effect due to the small number of targets presented for this task, since the overall scores for character presence were relatively high, therefore not leaving much possibility to improve. An issue that should be addressed in future research.

In addition, the increase in accuracy from the first to the fourth block (i.e., the effect of block number) could be due to the practice with the task. In fact, in addition to gaining familiarity with the task itself, participants could have developed personal strategies to perform the task in more efficient ways. For example, they could have learned that exactly four distractors followed every target, so when they were presented with the fourth distractor after a target, they “prepared” for the presentation of a new target by shifting their attention toward an encoding process even before the target appeared.

Let us now consider the average accuracy in correctly remembering the targets by their presentation order. The first target presented in each trial is remembered more correctly than the second, the second more than the third, and the third more than the fourth. This is commonly known as the primacy effect [46]. Such an effect was used by Glanzer and Cunitz [46], as evidence for a short-term storage of information functionally distinct from long-term storage. In case we had observed a difference in the magnitude of this effect between the two conditions, with a higher performance in the experimental condition, we could have interpreted it as evidence of the effect of cognitive load on information transfer to long-term memory, but this is not the case. In the context of the TBRS model of working memory, this effect can be explained by the fact that the memory trace of an item had more occasions to be refreshed compared to those items presented later, but less time than those presented before. This effect can be interpreted as a reflection of different levels of decay of memory traces held in working memory. Still, an alternative interpretation can be given by the different levels of encoding of memory traces in long-term memory. The delay between the presentation of the last target and the response phase should have, at least in theory, updated the content of working memory, substituting the target memory traces with the n-back task-relevant memory traces that changed after each new stimulus. If targets were maintained entirely in working memory, this means that the cognitive resources available to participants were enough to both process and update the new information from the n-back task and maintain the targets from the reading digit span task, but in this case, we might expect that the proportion of the activation of the target memory traces would have balanced over time. On the other hand, if participants did not have enough available cognitive resources to process and update n-back information while maintaining reading digit span task targets, and information was transferred to long-term memory during the processing and maintenance of the memory traces, it would be reasonable to expect that targets presented at the beginning of the series were better encoded in long-term memory, and, therefore, better recalled than the targets presented at the end. However, this interpretation needs to be corroborated further in future studies.

As already said above, our findings show a significant increase in accuracy from the first to the fourth block of trials, suggesting some possible effect of cognitive load. However, this improvement in performance could also be due to some practice effect. Either because participants got more familiar with the task (e.g., knowing that every four blue letters, a red one will appear) or because they became better at managing cognitive resources (for example, by finding strategies to cluster stimuli), or both. However, such explanations alone would not justify the difference in performance between the experimental and control conditions, which was overall higher in the experimental condition.

The unexpected difference in performance for remembering the targets’ presence and order should be further explored in a new study, in which the two aspects of the series might be experimentally controlled. We suggest that a paradigm with two separate conditions should be designed in order to test the performance for each of these two aspects separately. In fact, by designing an experimental paradigm that allows participants to only process one of the two aspects (item presence and item order) at a time, it will be possible to observe the two processes separately. Another unexpected observation was the absence of interaction between condition and trial. The absence of such an interaction might be due to a multitude of different factors, but the evidence collected in this study does not allow us to formulate any valid explanation without being too speculative. This is an issue that should be addressed by future studies specifically designed to investigate this element.

Interestingly, a clear primacy effect for the order of the presentation of characters was also found, and it can be interpreted as a proxy of the transfer of information from working memory to long-term memory.

### Limits of This Study

While this study provides useful insights into the role of cognitive load in information transfer and storage, the paradigm used does not allow a precise quantification of the variation in cognitive load among the different experimental trials. In other words, we know that the cognitive load in trial 2 is lower than in trial 1 and higher than in trial 3, but we cannot quantify such a difference. In addition, we do not know what type of mathematical function cognitive load follows in its decrease across trials. Therefore, we cannot say whether the difference in cognitive load between trial 1 and trial 2 is the same or not as the difference between trial 2 and trial 3. A new paradigm should be designed to take into consideration this element in a future study; in fact, a greater level of control on cognitive load would lead to a deeper understanding of its role in information transfer to long-term memory.

## 5. Conclusions

In conclusion, we found some evidence supporting the role of the cognitive load and its effect on the amount of information transferred from working memory to long-term memory. Interestingly, it also emerged that the processes underlying remembering a certain number of items and their specific serial order might be two distinct processes. This hypothesis is not new in the literature, but still needs evidence and testing, and we will investigate this possibility in a future study.

## Data Availability

The original data presented in the study are openly available in Bicocca Open Archive Research Data at https://board.unimib.it/datasets/r8b9xwgb7p/1, accessed on 27 October 2025.
